# Penalized factorial regression as a flexible and computationally attractive reaction norm model for prediction in the presence of GxE

**DOI:** 10.1007/s00122-025-04865-4

**Published:** 2025-03-28

**Authors:** Vahe Avagyan, Martin P. Boer, Junita Solin, Aalt D. J. van Dijk, Daniela Bustos-Korts, Bart-Jan van Rossum, Jip J. C. Ramakers, Fred van Eeuwijk, Willem Kruijer

**Affiliations:** 1 Biometris, Wageningen University and Research, Wageningen, The Netherlands; 2 Bioinformatics, Wageningen University and Research, Wageningen, The Netherlands; 3 Swammerdam Institute for Life Sciences, University of Amsterdam, Amsterdam, The Netherlands; 4 Faculty of Agricultural Sciences, Universidad Austral de Chile, Valdivia, Chile

## Abstract

**Key message:**

Penalized factorial regression offers a computationally attractive alternative to kernel and deep learning methods for prediction of genotype by environment interactions. For two representative data sets on wheat and maize, prediction accuracies were comparable, while computing requirements and time were clearly lower.

**Abstract:**

A longstanding challenge in plant breeding and genetics is the prediction of yield for new environments in the presence of genotype by environment interaction ($G \times E$). The genotypes in this case are promising candidate varieties at an advanced stage of breeding programs or are part of statutory variety trials or post registration trials. The genotypes have been tested in a limited set of trials and the question is how these genotypes will perform in future growing conditions. A reaction norm approach seems adequate to address this challenge. Reaction norms are functions with genotype-specific parameters that express the phenotype as a function of environmental inputs. $G \times E$ follows from differences in genotype-specific slope or rate parameters. Prediction of yield for new environments requires the identification of suitable reaction norm functions and the estimation of genotype-specific parameters together with knowledge about the environmental conditions. Here, we present penalized factorial regression with simple linear reaction norms for individual genotypes whose slopes are regularized by imposing a penalty upon them. Different types of penalization provide shrinkage, automatic selection of environmental covariates (*EC’s*) and protection against overfitting for prediction of yield with medium to large numbers of *EC’s*. Illustrations of our approach are given for a maize and a wheat data set. For these data, our approach compares well to alternative methods based on Bayesian regression and deep learning with respect to prediction accuracy, while computational demands are clearly lower.

**Supplementary Information:**

The online version contains supplementary material available at 10.1007/s00122-025-04865-4.

## Introduction

Genomic prediction has become an integral part of plant breeding (Voss-Fels et al. 2019). On a wide scale, molecular markers are used to improve the efficiency of breeding strategies. This efficiency gain follows from the fact that selection on the basis of genomic predictions, or genomic selection, permits shorter generation time and higher selection intensity. The genomic prediction model is created for a target phenotypic trait by estimating marker effects on a training set of genotypes with observed phenotypic values, and these estimated effects are then used to predict the phenotypic values for genotypes for which only marker information is available. A wide collection of prediction models, both statistical and machine learning models, is available (Crossa et al. 2017), the most popular one being the GBLUP model. The latter is a mixed model with random genotypic effects that are structured by a marker-based relationship matrix. The GBLUP model can be proven to be equivalent to a ridge regression on the full set of markers (Piepho et al. 2012).

For traits with strong Genotype-by-Environment interactions ($G \times E$), genomic prediction models will need to be extended to meet the requirement of genotypic effects being dependent on the environmental conditions. Two major classes of models are distinguished: character state models and reaction norm models (Falconer 1990; Dekkers 2021). In character state models, the same trait as observed in different environments is treated as if it were multiple traits, i.e., characters, between which genetic correlations need to be modelled. In reaction norm models, phenotypes for individual genotypes are functions of continuous environmental covariates (*EC’s*) and genetic correlations between environments are quadratic functions of the differences in *EC* values. Character state and reaction norm models are equivalent for the environments for which phenotypic observations and *EC* values are available, but it will be obvious that reaction norm models are more flexible in that they can provide predictions of phenotypes for environments for which no phenotypes were observed, but for which *EC* values are present.

This paper focuses on reaction norm models for the prediction of a phenotypic trait in new conditions, test conditions, for a collection of genotypes for which the trait in question was observed in a training set of trials. This is a prediction scenario that is pertinent for phenotypic evaluations of promising genotypes or candidate varieties in the last stages of breeding programs and in official variety trials testing for Value for Cultivation and Use (VCU), see Kempton et al. (2012). The training set of trials is typically part of a multi-environment trial, and both the training and test sets are intended to represent the future growing conditions of the genotypes within the target population of environments (Cooper and Hammer 1996; Chapman et al. 2000; Cooper et al. 2023). This scenario was labelled $G_{o}E_{n}$ by Bustos-Korts et al. (2016) and Malosetti et al. (2016), where the *G* stands for genotypes and the *E* for environments, with the subscript *o* indicating ‘observed’ genotypes and/or environments and the subscript *n* indicating ‘new’ genotypes and/or environments. Four scenarios were described by Bustos-Korts et al. (2016) and Malosetti et al. (2016), with $G_{o}E_{o}$ being the simplest, i.e., imputation in a two-way table of $G \times E$ means, and $G_{n}E_{n}$ being the most challenging, i.e., phenotypic prediction for non-observed genotypes in new environments, a kind of extrapolation.

Factorial regression models are a class of reaction norm models for the analysis of $G \times E$ data (Denis 1988, 1991; Van Eeuwijk et al. 1996). They are fixed two-way analysis of variance models with genotypic and environmental covariates determining contrasts on the levels of the genotypic and environmental factors. A genotype *i* has a linear response $v_{j}\beta _{i}$ to an *EC* *v* in environment *j*, $\beta _{i}$ being the genotype’s sensitivity. Early illustrations of this approach appear in Baril et al. (1995); Vargas et al. (1999); Voltas et al. (1999); Brancourt-Hulmel et al. (2000) and Brancourt-Hulmel and Lecomte (2003). Fixed factorial regression models become costly in terms of parameters to be fitted when the numbers of genotypes and *EC’s* increase. As a consequence, attempts have been made to replace the fixed regressions in factorial regression by random regressions. Examples of a random regression approach to $G \times E$ in plant breeding are Ly et al. (2018); Buntaran et al. (2021) and Rebollo et al. (2023). The issue with random regression for $G \times E$ is that it seems that few *EC’s* can be incorporated simultaneously without the mixed model fit becoming problematic. This problem will occur when we want our model to be translation and scale invariant. In random regression models for $G\times E$, each additional EC then requires a new variance component for the genotypic slope associated with that *EC*, along with covariance components between the intercept and the new slope, as well as between the existing slopes and the new slope. Interesting solutions for the formulation of random regression models with moderate to high numbers of *EC’s* for $G \times E$ were recently proposed by Tolhurst et al. (2022) and Piepho and Blancon (2023). Both papers propose rank reduction approaches to retain invariance. Outside applications in these seminal papers, these methods do not seem to have been applied to other data yet.

A nowadays popular reaction norm approach in a mixed model framework that is able to incorporate very large number of *EC’s* was presented by Jarquin et al. (2014). For the environments, the values on the *EC’s* define an environmental relationship matrix that imposes a correlation structure on those environments. The inclusion of an environmental relationship matrix, or kernel, on the random $G \times E$ effects in a linear mixed model produces the equivalent of a ridge regression formulation for $G \times E$ with genotypes differing in sensitivity to *EC’s* with the random slopes (sensitivities) subject to a common penalty (ratio of variance components). In addition to imposing a relationship matrix on the environments, Jarquin et al. (2014) also imposes a relationship matrix on the genotypes. When this genotypic relationship matrix is based on molecular markers, the model becomes a genomic prediction model accounting for $G \times E$ , where the variance-covariance matrix for the $G \times E$ effects is structured by the Kronecker product of the genotypic and environmental kernel.

An important reason for the popularity of the Jarquin et al. (2014) model is its implementation in the R-package BGLR (Perez and de los Campos 2014). The fitting of the model within the Bayesian context of BGLR | is, however, computationally demanding. The purpose of this paper is to present a reaction norm approach to $G \times E$ that allows the inclusion of large numbers of *EC’s*, comparable to the approach by Jarquin et al. (2014), but with less computational effort. Our approach addresses especially the $G_{o}E_{n}$ prediction scenario without requiring markers. We introduce a penalized form of factorial regression with a choice for the type of penalty by the user. For each genotype, an intercept and multiple slopes are estimated on a training set of genotypes and environments. The estimated intercepts and slopes are transferred to a test set of new environments for which the values of the *EC’s* are assumed to be known. The latter assumption is a common simplification. A possible work-around was presented by Gillberg et al. (2019) and Los Campos et al. (2020), who used historical distributions of *EC’s* as priors in Bayesian prediction models. In the remainder of this paper, we will first describe the penalized factorial regression model and the estimation of its parameters, followed by an illustration of our approach to maize data from the EU-DROPS project (Millet et al. 2016, 2019) and another illustration on APSIM simulated wheat data with more details in Bustos-Korts et al. (2019) and Bustos-Korts et al. (2020). We developed the R-package factReg to fit our penalized factorial regression models. We compare our penalized factorial regression approach with a model following the established method of Jarquin et al. (2014) and a deep learning proposal (Khaki and Wang 2019).

## Materials and methods

### Notation

We assume observations $Y_{i,j}$ of a phenotypic trait *Y* for genotypes $i=1,\ldots ,n_g$ in environments $j=1,\ldots ,n_e$, and $n_c$ environmental covariates (*EC’s*) with values $v_{i,j}^{(t)}$ ($t = 1,\ldots ,n_c$). Here, $n_g$ and $n_e$ are the numbers of training genotypes and training environments, respectively. The number of training environments should be large enough to adequately estimate genotypic sensitivities, which depends on the number of *EC’s* and model complexity.

In addition to these training environments, there is a number of test environments, for which only *EC’s* are available. Our main objective is to obtain high ‘per environment’ accuracies for the test environments, i.e., high correlation (across genotypes) between predicted and observed values. Often, environments are grouped into environmental scenarios (or envirotypes), see Millet et al. (2019). Accurate prediction in the test environments is only possible when the scenarios present in the test environments are also observed among the training environments.

The *EC’s* can have environment dependent values that are the same across all genotypes in an environment, $v_{j}^{(t)}$. However, it is equally possible for the *EC’s* to have genotype-specific *EC* values, i.e., $v_{i,j}^{(t)}$, with the *EC* having a unique value for each combination of genotype and environment. This genotype specificity occurs for covariates adapted to each genotype’s developmental stage. For example, maximum daily temperature around flowering time may differ between early and late flowering genotypes. In some of the models below (in particular the factorial regression model), the interpretation of main effects is different when *EC’s* have a constant value within an environment, i.e., all genotypes share the same *EC* value, but this does not matter much for our purpose of prediction, in the $G_{o}E_{n}$ scenario.

### Maize and wheat data

We evaluate the different prediction methods on two multi-environment data sets, one experimental and the other one simulated. The experimental data belonged to a $G_{o}E_{n}$ problem in maize studied by Millet et al. (2019), with 246 maize hybrids evaluated in 25 trials (environments) that will serve as training data. All genotypes occurred in all trials in the training set. The training data set was fully balanced. The test set consisted of 12 independent trials in which a subset of 32 hybrids out of the full set of 246 hybrids was evaluated. There were $n_c=11$ *EC’s* with genotype-specific values, i.e., with unique values for each genotype by environment combination, reflecting the growing conditions for these maize hybrids. With respect to these growing conditions, five environmental scenarios (envirotypes) were defined: two water-deficit scenarios (‘WD-Hot (day)’ and ‘WD-Hot’) and three well-watered scenarios (‘WW-Cool’, ‘WW-Hot(day)’ and ‘WW-Hot’). In the test environments, only the well-watered scenarios were represented (as in Millet et al. (2019)). All 246 hybrids were genotyped with 333k SNPs, see again Millet et al. (2019).

For our second example, we used wheat data generated by the Agricultural Production Systems Simulator, or APSIM (Keating et al. 2003; Holzworth et al. 2014). These data were generated on the basis of historical weather and soil information for the period 1983-2013 for four locations in Australia. In contrast to the earlier papers by Bustos-Korts et al. (2019) and Bustos-Korts et al. (2020), two sowing times per year by location combination were included instead of one. The total number of trials with yield data was 248. The set of 40 environments corresponding to the trials after 2008 was chosen as the test set. Both training and test set were fully balanced in that all genotypes occurred in all trials. More details on the APSIM simulation settings are given by Bustos-Korts et al. (2019) and Bustos-Korts et al. (2020). The data for the statistical analyses in the current paper included 160 genotypes, which were randomly selected from a total of 199 genotypes present in Bustos-Korts et al. (2020). For the genotypes, 3035 SNP markers were available. Yield data generated by APSIM do not contain an experimental error. To mimic a trait with medium sized heritability, an error was added with environment specific variance, such that heritabilities (within environments) were around 0.5. There were in total $n_c=57$ genotype-specific *EC’s*, i.e., between 10 and 12 *EC’s* for each of five stages in the growing season, and sowing date (Bustos-Korts et al. 2016, see for more details). For these simulated wheat data, an environmental characterization was done that resulted in four envirotypes (Keating et al. 2003; Chenu et al. 2013; Bustos-Korts et al. 2019, 2020). The envirotypes were labelled as ET1, ET2, ET3 and ET4. All envirotypes occurred in training and test set. With respect to these envirotypes, training and test set were both reasonably balanced with about equal frequencies of occurrence for the four envirotypes. ET1 contained environments that did not suffer from water stress. In contrast, plants in ET4 did suffer from severe water stress that started early in the growing season, without stress relief. Type ET2 contained environments with post-flowering mild stress. Finally, ET3 corresponded to water stress that started early in the season and was relieved during the grain filling.

### Penalized factorial regression

**Table 1:** Parameter values for Deep Learning models: *NL* (the number of layers), *NN* (the number of neurons), $\xi$ (the learning rate), and the regularization parameters $g_1$ (for the first layer) and $g_2$ (for all other hidden layers)

	Method	NL	NN	$\xi$	$g_1$	$g_2$
Maize data	Deep Learning M1	21	50	0.0003	0.6	0.2
	Deep Learning M2	21	50	0.0003	0.06	0.02
	Deep Learning M3	3	50	0.0003	0.0006	0.0002
Wheat data	Deep Learning W1	11	3	0.0003	0.0006	0.0002
	Deep Learning W2	11	3	0.003	0.0006	0.0002
	Deep Learning W3	11	50	0.0003	0.0006	0.0002
	Deep Learning W4	11	50	0.003	0.0006	0.0002

**Table 2:** Average prediction accuracy for training and test environments ($\text {APCOR}_{\text {Env}}$), for the maize data

Model	Training set	Test set
Additive	0.716	0.541
Additive with three covariates (Millet et al. 2016)	0.797	0.591
Marker-EC (Jarquin et al.)	0.935	0.585
BRR	0.831	0.584
Deep Learning M1	0.680	0.464
Deep Learning M2	0.679	0.353
Deep Learning M3	0.561	0.176
Full (factReg with$\lambda =0$)	0.899	0.349
FactReg (El-Net)	0.848	0.576
FactReg (LASSO)	0.848	0.576
FactReg (ridge)	0.824	0.586

**Table 3:** Average prediction accuracy for training and test environments ($\text {APCOR}_{\text {Env}}$), for the wheat data

Model	Training set	Test set
Additive	0.610	0.593
Marker-EC (Jarquin et al.)	0.879	0.779
BRR	0.844	0.794
Deep Learning W1	0.508	0.555
Deep Learning W2	0.821	0.814
Deep Learning W3	0.888	0.850
Deep Learning W4	0.892	0.822
Full (factReg with $\lambda =0$)	0.828	0.707
FactReg (El-Net)	0.856	0.819
FactReg (LASSO)	0.857	0.813
FactReg (ridge)	0.843	0.779

**Table 4:** Computational time for *factReg* and competing models. Deep Learning was performed on an Intel Xeon Silver 4116 CPU, 2.10 GHz, with 12 cores. Because of the large memory requirements, we used Intel Xeon E5-1650, 3.6 GHz, for the Jarquin et al. model for the wheat dataset. All other analyses were performed on an Intel Xeon W-2235 CPU, 3.8 GHz

Model	Maize data	Wheat data
factReg	2 sec	22 sec
Deep Learning	$\sim$ 1 hour	15 - 25 min
BRR	24 min	$\sim$ 7 hours
Marker-EC (Jarquin et al. 2014)	$\sim$ 3 hours	$\sim$ 6 days

Penalized factorial regression assumes a model of the form

1 $$\begin{aligned} Y_{i,j} = \mu + g_i + e_j + \sum _{t=1}^{n_c} v_{i,j}^{(t)} \beta _{i,t} + \epsilon _{i,j}, \end{aligned}$$

where each genotype *i* has sensitivities $\beta _i=(\beta _{i,1},\ldots ,\beta _{i,n_c})$, quantifying its response to changes in the set of $n_c$ covariates. These sensitivities will be penalized, as we will explain in the next section. $\mu$ is the general mean, and $g_i$ and $e_j$ are genotypic and environmental main effects, respectively. The errors $\epsilon _{i,j}$ are assumed to be independent and identically distributed (i.i.d.) Gaussian with constant variance $\sigma ^2$. Here, *t* denotes the index of the environmental covariates. To make the model identifiable we set the main effects of the first environment and of the first genotype to zero, i.e., $e_1 = 0$ and $g_1 = 0$ (identifiability in factorial regression is discussed in more detail in Denis (1988)). When we assume that *EC* values are available for new (unobserved) environments and that the genotypic main effects and slopes estimated in the training set can be transferred to the test set, we can use model (1) to predict the environment-centred yield for new environments. Estimates for $g_i$ and $\beta _i$ in the training set are thus combined with *EC* values for test environments. Prediction accuracies can be calculated from the Pearson correlation between observed and predicted yields for the test environments, estimates for the environmental main effects for the test environments are not necessary for that. To convert the factorial regression model into a genomic prediction model, we can follow the strategy introduced in Millet et al. (2019). The genotypic main effects and slopes are estimated by factorial regression first and subsequently these estimates are regressed on marker profiles. Here our main objective is an efficient methodology for prediction in the $G_{o}E_{n}$ scenario, in which case the step of regressing the genotypic parameters on markers becomes optional. We will focus on the development of a penalized factorial regression framework without the use of markers unless stated otherwise.

#### Penalization

Equation (1) defines a linear model with in total $n_g + n_e - 1 + n_g n_c$ parameters, i.e., $n_c+1$ parameters *for each* genotype (Appendix A provides an example of a design matrix). Consequently, given the large numbers of genotypes and *EC’s* that can be measured nowadays, the number of parameters is typically similar to or larger than the number of observations, requiring some form of penalization. Here we use ridge penalization with the form $\dfrac{\lambda }{2} \Vert \beta \Vert _2^2$ (Hoerl and Kennard 1970). For comparison, we will also report the accuracies for LASSO (Least Absolute Shrinkage and Selection Operator) penalty, defined as $\lambda \Vert \beta \Vert _1$ (Tibshirani 1996), and Elastic Net penalty, defined as $\lambda \left( \alpha \Vert \beta \Vert _1 + \dfrac{1-\alpha }{2} \Vert \beta \Vert _2^2\right)$ (Zou and Hastie 2005). Here, the parameter $\lambda$ is the amount of penalization and was determined through cross-validation. We investigated two schemes for cross-validation process: random and stratified. The first scheme was a random cross-validation in which the default of 10-fold cross-validation was implemented. Partitioning was based on the $G \times E$ observations, ignoring the environments. In case of stratified cross-validation, the environment structure is taken into account. In our study, we found stratified cross-validation for the maize data to work best and random cross-validation for the wheat data.

For this paper, we chose an elastic net implementation with equal weights ($\alpha =0.5$) on LASSO and ridge penalties. We provide further details in Appendix A. We chose not to penalize the genotypic and environmental main effects in model (1). The environmental main effects, in particular, are typically much larger than the $G \times E$ that we aim to capture with *EC’s*. Equal penalization for main effect and interaction parameters will then lead to too strong shrinkage for the main effects and too weak shrinkage for the genotypic sensitivities in the $G \times E$ part of the model.

#### Software

We implemented penalized factorial regression in the new R-package factReg (Kruijer et al. 2023), available from CRAN. The main function is GnE(), which makes predictions based on penalized factorial regression for the prediction scenario $G_{o}E_{n}$ using the glmnet package (Friedman et al. 2010). Computation times were sped up by the use of the sparse matrix facilities of glmnet() function. For example, the relatively large wheat data set with 160 genotypes and 208 training environments took less than half a minute on a standard desktop. More details on computation times are given in the Results section (see Table 4 below).

### Additive baseline

To evaluate the success of our $G \times E$ models, we consider the additive model with main effects for genotype and environment and without a $G \times E$ term:

2 $$\begin{aligned} Y_{i,j} = \mu + g_i + e_j + \epsilon _{i,j}. \end{aligned}$$

Model (2) is like model (1) but without the terms involving *EC’s*.

### Standard Bayesian random regression (BRR)

By interpreting the sensitivities in model (1) as independent Gaussian random effects with common variance we obtain the random regression equivalent of penalized factorial regression (with ridge penalty). We obtain parameter estimates and predictions using a fully Bayesian approach, using the default priors in the R-package BGLR ( Perez and de los Campos (2014), version 1.1.0), running the MCMC chain for 50,000 iterations, including a burn-in of 10,000 and a thinning rate of 5.

### Bayesian regression with $marker \times \textit{EC}$ and $marker \times environment$ interactions

The Bayesian random regression models mentioned above do not use any marker information. Jarquin et al. (2014) proposed models with two types of random $G \times E$ effects. Firstly, $G \times E$ effects that are the result of marker-*EC* interactions (*gw*), and secondly, residual $G \times E$ effects that cannot be explained by *EC’s* (*ge*). Following Jarquin et al. (2014) we assume the model

3 $$\begin{aligned} Y = \mu \cdot 1_N + Z_E e + Z_G g + (gw) + (ge) + \epsilon , \end{aligned}$$

where $\mu$ is the intercept, *N* is the total number of observations, $Z_E$ and $Z_G$ are the design matrices for the environmental and genotypic main effects. Given a genetic relatedness matrix *K*, the vector *g* of genotypic main effects has a $N(0, \sigma _g^2 K)$ distribution, whereas the environmental main effects contained in *e* are assumed independent (alternatively their dependence can be modelled using an environmental relatedness matrix). Let *W* be the $N \times n_c$ matrix containing the standardized *EC’s*. The vector (*gw*) is multivariate normal with covariance $(Z_G K Z_G^t) \circ (WW^t)\sigma _{gw}^2$, where $\circ$ denotes the Hadamard (cell-wise) product. Similarly, the vector (*ge*) has covariance $\left( {Z_{G} KZ_{G}^{t} } \right) \circ \left( {Z_{E} Z_{E}^{t} } \right)\sigma _{{ge}}^{2}$. This vector represents $marker \times environment$ interactions. To obtain predictions from model (3) we used BGLR, with same settings as in Sect. Standard Bayesian random regression (BRR).

### Deep learning with feed-forward networks

We include a comparison with deep learning-based multi-environment genomic prediction, for which various proposals exist in the literature (Montesinos-López et al. 2021; Ma et al. 2014; Mochida et al. 2019; van Dijk et al. 2021). Here we take the feed-forward network described by Khaki and Wang (2019), winning the 2018 Syngenta Crop Challenge. This model requires *EC’s* as well as marker data, which are used as separate inputs each with their own neurons. The neural network code was implemented in Python using the Tensorflow library. Stochastic gradient descent was used with a mini-batch size of 64. Batch normalization was used before activation for all hidden layers except the first hidden layer. As described in detail in Khaki and Wang (2019), maxout activation was used, as well as LASSO regularization (on the first hidden layer) and Ridge regularization (for all other hidden layers).

We considered varying values for the following five hyper parameters: the number of layers *NL*, the number of neurons *NN*, the learning rate $\xi$, and the regularization parameters $g_1$ (for the first layer) and $g_2$ (for all other hidden layers). From different combinations of these values, we have selected those which provided the best prediction accuracy on testing data. The considered values of these parameters are shown in Table 1. We will report the performance of the Deep Learning model with the highest average prediction accuracy, $\text {APCOR}_{\text {Env}}$, for the test set. This might lead to overfitting, given that we do not cross-validate within the training set. This would be more computationally costly and with our current setup we are rather conservative in claiming that the penalized factorial regression prediction has a higher accuracy (given the potentially too optimistic performance reported for the Deep Learning model).

### Evaluation of accuracies

Throughout the paper we focus on the Pearson correlation (*r*) within each environment as criterion for prediction accuracy. For the maize data we consider the correlation between predicted and observed phenotypic values, and for the wheat data the correlation between predictions and simulated genetic effects. In addition, we use the Pearson correlation averaged over environments ($\text {APCOR}_{\text {Env}}$). We provide the mathematical expression for this accuracy measure in the Supplementary Materials (see Appendix B).

## Results

**Fig. 1:**
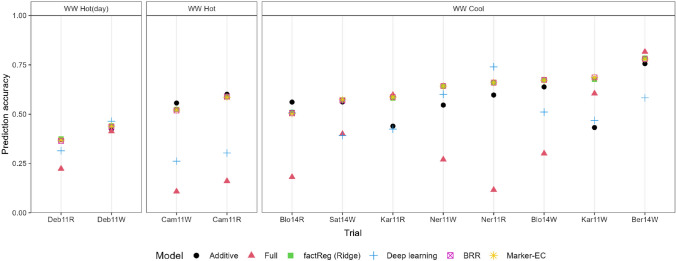
Accuracies for the test environments, maize data. The environments are ordered based on the average accuracy

**Fig. 2:**
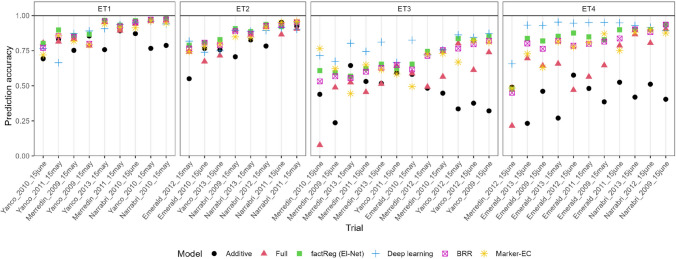
Accuracies for the test environments, wheat data. Trials are classified into four environmental groups (ET1-ET4) based on the time and severity of water stress. The environments within clusters are ordered based on the average accuracy

Accuracies (Pearson correlations) for maize are shown in Fig. 1 and Table 2, while those for wheat appear in Fig. 2 and Table 3. The accuracies in Figs. 1 and 2 pertain to the test environments. For Deep Learning, these figures show the model with highest average prediction accuracy ($\text {APCOR}_{\text {Env}}$). For the maize test data, average accuracy was highest for penalized factorial regression (0.586 with ridge penalty, and 0.576 with LASSO and elastic net). BRR had almost the same accuracy (0.584) as the Jarquin et al. model (i.e., model (3) with marker-*EC* interactions) (0.585). There was some difference between these methods across environments. Inspecting Fig. 1, non-penalized factorial regression clearly did worse than penalized factorial regression, the Jarquin et al. model and deep learning. Deep learning had trouble predicting ‘WW hot’ environments. For the maize data, the computational time for penalized factorial regression was two seconds, whereas for BRR model it was 24 minutes and for model (3) it was more than three hours (see Table 4). Deep learning performed poorly, possibly because of the too small sample size at the side of the environments. Moreover, it took several hours to fit the models M1 and M2 (which is partly due to large number of markers).

For the wheat data, the deep learning models performed better than for the maize data (the best architecture, W3, provided an average accuracy for the test data of 0.850). The performance of penalized factorial regression with elastic-net or LASSO penalty was the next best (with average accuracies of 0.819 and 0.813, respectively), closely followed by penalized factorial regression with ridge penalty (0.779). Differences between these methods were mostly small in environment types ET1 and ET2, but differences were larger in ET3 and ET4, which contained environments with severe drought stress. The computational time for penalized factorial regression was around 20 seconds, whereas for deep learning it was around 20 minutes. Again, BRR achieved almost equal accuracies as penalized factorial regression (0.794), however it took over seven hours to run. The $marker \times \textit{EC}$ model (3) had average accuracy 0.778, but it took more than six days to run, requiring more than 100 Gb of RAM.

On average, penalized factorial regression and Bayesian approaches (with or without marker-*EC* interactions) all outperformed the additive model, in both data-sets. In other words, including the *EC’s* generally improved prediction accuracy in both training and test environments. Genotypic main effects were however rather large, and for this reason there were several environments, where the additive model had reasonable accuracy; in some cases it was even among the best methods.

**Fig. 3:**
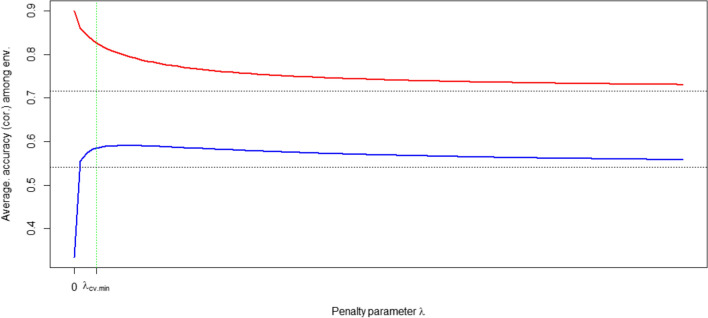
$\text {APCOR}_{\text {Env}}$ of *factReg* (ridge) model for the test (blue) and for the train (red) environments, maize data, for different levels of the penalty parameter $\lambda$. Dotted horizontal lines correspond to the average accuracies for the Additive model

Including all covariates without penalization clearly led to overfitting, with accuracies being higher in the training environments but lower in the test environments. These results are shown in Fig. 3 for the maize data, which shows average prediction accuracy ($\text {APCOR}_{\text {Env}}$) of penalized factorial regression as function of the penalty parameter $\lambda$. For larger values of $\lambda$, penalized factorial regression converges towards the additive model (dotted lines in Fig. 3). In addition, the estimated penalty parameter $\lambda _\text {cv.min}$ (selected through cross-validation) was close to its most efficient value in terms of the testing accuracy.

For the maize data, Millet et al. (2016) used a combination of physiological knowledge, prior insight in interactions between *EC’s*, and goodness-of-fit statistics to identify a candidate set of *EC’s* for prediction of $G \times E$: intercepted radiation early in the season (Ri.Early), night temperature around flowering time (Tnight.Flo), and soil water potential in the same developmental period (Psi.Flo). We estimated the parameters for a simple fixed factorial regression model with only these three *EC’s* on a training set of environments and achieved a prediction accuracy for the test set that was higher than any of the other prediction models we had constructed so far: 0.591 (Table 2). Apparently, it pays off to do subset selection on *EC’s* using biological insights. When we fitted all possible subsets of three *EC’s* out of the full set of 11 *EC’s* that were available, the prediction accuracies ranged from 0.404 to 0.611, with a median of 0.526 and a mean of 0.523. The *EC’s* identified by Millet et al. (2016) provided an accuracy that was almost as high as that of the best set of three *EC’s*. For the penalized factorial regression with LASSO penalty we expected the variance of the *EC* slopes to be related to the importance of the *EC* for predicting $G \times E$. These variances varied from 0.0677 for Tmax.Flo to 0.146 for Tnight.Early. The variances for Ri.Early, Tnight.Flo and Psi.Flo belonged to the intermediate ones with 0.116, 0.122 and 0.123, respectively. Therefore, the variation in penalized LASSO slopes across genotypes for an *EC* indeed seemed related to its importance for predicting $G \times E$. A heat plot for the LASSO slopes moderately supports the same conclusion (Supplementary Figure S1 in Appendix C).

## Discussion

Predicting the performance of genotypes in new environments is important in plant breeding but current approaches suffer from computational or statistical problems. Here we took a reaction norm perspective and extended the classical factorial regression model using a ridge, LASSO, or elastic net penalty on the estimated genotypic sensitivities. Fast estimation of the model parameters under these different types of penalties was possible with our specifically developed R-package factReg (Kruijer et al. 2023), which relies on functions inside the glmnet package (Friedman et al. 2010). When choosing a ridge penalty, our model is essentially the same as that in Jarquin et al. (2014), but the proposed estimation there follows a Bayesian protocol included in the R-package BGLR (Perez and de los Campos 2014) that is clearly more computation-intensive than our approach. A further, mixed model based, ridge penalty estimation procedure was presented by Tolhurst et al. (2022). The corresponding random regression model is in all cases relatively simple in that it assumes independence between genotypic intercepts and slopes and therefore lacks the property of translational invariance as was pointed out by Tolhurst et al. (2022) who also presents viable alternative modelling options for random regressions that do possess this property. It is worthwhile to recall that the use of prior biological knowledge to reduce the number of potentially interesting *EC’s* before any other modelling considerations are made produces a very high return on investment as illustrated by the comparatively high accuracy of the fixed factorial regression model. A similar conclusion on the usefulness of feature selection, EC selection, to improve prediction of $G \times E$ was reached by Montesinos-López et al. (2023).

Our approach has, of course, some limitations. As for any machine learning or statistical method, it is challenging to predict phenotypic responses for future growing conditions, where such conditions may be outside the current environmental conditions covered by the training set of environments (Bustos-Korts et al. 2021; Cooper et al. 2023). Among others, Robert et al. (2020), Malosetti et al. (2016) and Rogers and Holland (2022) show that prediction accuracies will increase when the conditions included in the training set of environments overlap with those of the test set of environments. One way to improve the environmental overlap between training and test set is by using an additional environmental characterization in which individual trials or environments are assigned to a number of discrete classes in terms of environmental scenarios or envirotypes. When the trials in both training and test belong to certain classes of environments, it can be verified relatively easily whether training and test set cover the same envirotypes or scenarios. It will be hard to predict performance under drought stress from a training set of trials that contained only well-watered trials. An application of this strategy occurs in (Millet et al. 2019).

Another limitation is that the current version of our implementation of penalized factorial regression focuses on prediction of yield in new environments for existing genotypes ($G_{o}E_{n}$), but does not cover new genotypes in new environments ($G_{n}E_{n}$). A straightforward way to arrive at predictions for the $G_{n}E_{n}$ scenario is by estimating genotypic slopes and intercepts on the existing (observed) set of genotypes and then use a standard uni- or multivariate genomic prediction method to regress the estimates for those intercepts and slopes on marker profiles. From this genomic prediction model genotypic intercepts and slopes can be produced for a set of new (unobserved) genotypes and subsequently these predicted genotypic intercepts and slopes can be combined with *EC* values to obtain yield predictions for the $G_{n}E_{n}$ scenario, like in Millet et al. (2019).

A point to consider then is that when the number of *EC’s* is large, many of the estimated sensitivities will be zero or very small, and different *EC’s* will be selected for different genotypes. Some of the individual *EC’s* will then have few informative values, leading to poor genomic predictions for the corresponding sensitivities. A possible remedy may be the use of group LASSO (Yuan and Lin 2006), which would enforce each *EC* being selected either for all genotypes simultaneously or for none.

Another possible limitation is that our penalized factorial regression cannot assess the amount of $G \times E$ interaction that is unexplained by the *EC’s*, but this would be relatively straightforward if we used plot-level data, as was recently proposed by Lopez-Cruz et al. (2023).

Our methodology can be extended in several directions. First, the penalty on sensitivities could be genotype-specific instead of being uniform across all genotypes. We experimented with this and our results suggest that when the number of environments is large enough, this extension leads to an improvement of the accuracies. For the wheat data this indeed leads to considerable differences in selected penalties, and small improvements in accuracy. For the maize data, however, the accuracies were lower, on average, due to the small number of training observations per genotypes (only 25). Similarly, it may be advantageous to penalize the genotypic main effects as well. Typically, the magnitude of these effects is intermediate, somewhere in between that of the large environmental main effects and smaller $G \times E$ effects. Therefore, shrinkage of genotypic main effects should be allowed to differ from shrinkage of $G \times E$ parameters.

Another extension would be the inclusion of nonlinear transformations of *EC’s*, for instance using quadratic and higher order terms, or splines (Mumford et al. 2023). Without any form of regularization this would, of course, quickly lead to overfitting. For example, fitting of a response surface model including linear, quadratic and cross product terms requires $1/2 n_c(n_c + 1)$ degrees of freedom for each genotype, with only $n_e$ observations (i.e., the number of environments). Moreover, the rule of thumb suggests at least 10 observations per parameter for an accurate prediction, although this number may increase for noisy data (Hair et al. 1998). Random regression models naturally impose shrinkage but still seem difficult to fit for larger numbers of *EC’s* (Buntaran et al. 2021). In our penalized regression framework on the other hand it is straightforward to include higher order terms, but this does not always lead to higher accuracy.

Linear mixed models and Bayesian equivalents have been the preferred choice for genomic prediction. Jarquin et al. (2014) presented a by now very popular generalization from single to multiple environment prediction using a reaction norm formulation for the $G \times E$ effects. Typically, no feature selection takes place within the set of *EC’s*. Our penalized factorial regression is close in spirit to the method described by Jarquin et al. (2014), but is computationally more attractive and does allow feature selection under a LASSO penalty.

A rather different and strongly biologically inspired approach to multi-environment genomic prediction under $G \times E$ was proposed by Technow et al. (2015). These authors offer a framework that consists of prediction by dynamic crop growth models that integrate environmental inputs over time to produce as output yield in a genotype and environment specific way, i.e., with $G \times E$. Crop growth models consist of modules that communicate and interact. State variables and rate parameters within those modules determine yield components. A necessary but not sufficient condition for $G \times E$ at the output level of yield is that a subset of the yield components is genotype-specific. The structure of the crop growth model, the way in which the modules communicate and interact with each other, including feed forward and feed back loops, then defines the way environmental inputs will be integrated with genotype-specific yield components to become yield with the possibility of $G \times E$. The yield components themselves may still behave as simple additive functions of marker effects whose value can be predicted by genomic prediction. Promising applications of this crop growth model - whole genome prediction (CGM-WGP) approach were presented for maize by Messina et al. (2018) and for wheat by Jighly et al. (2023). No direct comparisons are available with respect to the prediction accuracy of on the one hand mixed and penalized regression models and on the other hand CGM-WGP. Mixed and penalized regression models have less strict requirements with respect to the nature, measurement time and frequency, and scale of *EC’s* and require less biological modelling skills. These statistical models, however, might be less successful at the edges and beyond their environmental training data domain.

An interesting compromise was recently introduced by Robert et al. (2020): crop growth model- trait-assisted prediction (CGM-TAP). In CGM-TAP, secondary phenotypic traits that are related to the target trait are measured in the training set alongside with the target trait, but in the test set (or selection set) the secondary trait is calculated by a crop growth model. Encouraging results were obtained by Robert et al. (2020) in bread wheat with yield as target trait and heading date as secondary trait. Each of the above described methods, mixed models as defined by Jarquin et al. (2014), CGM-WGP and CGM-TAP offers interesting options for multi-environment genomic prediction, but will require considerably more prior considerations combined with higher computational efforts than our penalized factorial regression. Therefore, we feel that our method provides a convenient and easy to use tool to investigate the predictive power of a set of *EC’s* in a multi-environment context with $G \times E$ that can be used as a first step in building more complex prediction models, but equally so can provide a satisfactory end model for prediction.

## Supplementary Information

Below is the link to the electronic supplementary material.

Supplementary file 1 (pdf 239 KB)
