# Supplementary material for: Penalized factorial regression as a flexible and computationally attractive reaction norm model for prediction in the presence of GxE
Source: Theor Appl Genet. 2025 Mar 28;138(4):88. doi: 10.1007/s00122-025-04865-4 (PMC11953130; doi:10.1007/s00122-025-04865-4)

Supplementary Material: Penalized factorial regression as a flexible and computationally attractive reaction norm model for prediction in the presence of GxE

Appendix A: Methodology

We have considered the following factorial regression

$$Y_{i,j} = \mu + g_i + e_j + \sum_{t=1}^{n_c} v_{i,j}^{(t)} \beta_{i,t} + \epsilon_{i,j}, \tag{S1}$$

We rewrite the factorial regression model (S1) in following matrix form:

$$
\mathbf{Y} = \begin{pmatrix} Y_{1,1} \\ Y_{1,2} \\ \vdots \\ Y_{n_g,n_e-1} \\ Y_{n_g,n_e} \end{pmatrix} = \begin{pmatrix} \mu \\ \mu \\ \vdots \\ \mu \\ \mu \end{pmatrix} + \begin{pmatrix} g_1 \\ g_1 \\ \vdots \\ g_{n_g} \\ g_{n_g} \end{pmatrix} + \begin{pmatrix} e_1 \\ e_2 \\ \vdots \\ e_{n_e-1} \\ e_{n_e} \end{pmatrix}
$$

$$
+ \begin{pmatrix} V_{1,1}\beta_1 \\ V_{1,2}\beta_1 \\ \vdots \\ V_{n_g,n_e-1}\beta_{n_g} \\ V_{n_g,n_e}\beta_{n_g} \end{pmatrix} + \begin{pmatrix} \epsilon_{1,1} \\ \epsilon_{1,2} \\ \vdots \\ \epsilon_{n_g,n_e-1} \\ \epsilon_{n_g,n_e} \end{pmatrix}
$$

$$
= \begin{pmatrix} 1 \\ 1 \\ \vdots \\ 1 \\ 1 \end{pmatrix} \mu + \begin{pmatrix} 1 \; 0 \; \ldots \; 0 \\ 1 \; 0 \; \ldots \; 0 \\ \vdots \\ 0 \; 0 \; \ldots \; 1 \\ 0 \; 0 \; \ldots \; 1 \end{pmatrix} \times \begin{pmatrix} g_1 \\ g_2 \\ \vdots \\ g_{n_g-1} \\ g_{n_g} \end{pmatrix}
$$

$$
+ \begin{pmatrix} 1 \; 0 \; \ldots \; 0 \; 0 \\ 0 \; 1 \; \ldots \; 0 \; 0 \\ \vdots \\ 0 \; 0 \; \ldots \; 1 \; 0 \\ 0 \; 0 \; \ldots \; 0 \; 1 \end{pmatrix} \times \begin{pmatrix} e_1 \\ e_2 \\ \vdots \\ e_{n_e-1} \\ e_{n_e} \end{pmatrix}
$$

$$
+ \begin{pmatrix} V_{1,1} \; 0 \; \ldots \; 0 \\ V_{1,2} \; 0 \; \ldots \; 0 \\ \vdots \\ 0 \; 0 \; \ldots \; V_{n_g,n_e-1} \\ 0 \; 0 \; \ldots \; V_{n_g,n_e} \end{pmatrix} \times \begin{pmatrix} \beta_1 \\ \beta_2 \\ \vdots \\ \beta_{n_g-1} \\ \beta_{n_g} \end{pmatrix} + \begin{pmatrix} \epsilon_{1,1} \\ \epsilon_{1,2} \\ \vdots \\ \epsilon_{n_g,n_e-1} \\ \epsilon_{n_g,n_e} \end{pmatrix}
$$

$$
= \mu\mathbf{1} + \mathbf{A}_1 g + \mathbf{A}_2 e + \mathbf{V}\beta + \epsilon
$$

$$
= \mu\mathbf{1} + [\mathbf{A}_1, \mathbf{A}_2, \mathbf{V}]\,(g', e', \beta')' + \epsilon
$$

$$
= \mu\mathbf{1} + \mathbf{X}\gamma + \epsilon,
$$

where we set e_1 and g_1 to zero, in order to guarantee the "identifiability" of the model. Here, $\mathbf{Y} \in \mathbb{R}^{n_{ge} \times 1}$ is a vector which contains all $Y_{i,j}$ yields, $\mathbf{X} =$

$[\mathbf{A}_1, \mathbf{A}_2, \mathbf{V}] \in \mathbb{R}^{n_{ge} \times (n_g - 1 + n_e - 1 + n_g n_c)}$ is a joined design matrix which contains the set of dummy variables corresponding to the genotypic and environmental main effects, and the set of environmental covariates per genotypes, $\mathbf{1} \in \mathbb{R}^{n_{ge} \times 1}$ is a column vector of ones, and $\epsilon \in \mathbb{R}^{n_{ge} \times 1}$ is a column vector which contains error terms $\epsilon_{i,j}$. Here, $\gamma = (g', e', \beta')' \in \mathbb{R}^{(n_g - 1 + n_e - 1 + n_g n_c) \times 1}$ is a column vector which contains all coefficients of interests, i.e., genotypic main effects $g = \left(g_2, ..., g_{n_g}\right)' \in \mathbb{R}^{(n_g - 1) \times 1}$, environmental main effects $e = (e_2, ..., e_{n_e})' \in \mathbb{R}^{(n_e - 1) \times 1}$ and sensitivities $\beta = \left(\beta'_1, ..., \beta'_{n_g}\right)' \in \mathbb{R}^{n_g n_c \times 1}$, where each $\beta_i \in \mathbb{R}^{n_c \times 1}$ are the genotype sensitivities, defined earlier.

Formally, we write the Elastic Net penalized solution of the coefficients as

$$\{\hat{\mu}, \hat{\gamma}\} = \arg\min_{\mu, \gamma} \frac{1}{2} \|\mathbf{Y} - \mu \mathbf{1} - \mathbf{X}\gamma\|_2^2 + \lambda \left(\alpha \|\beta\|_1 + \frac{1 - \alpha}{2} \|\beta\|_2^2 \right)$$

where $\| \cdot \|_2$ and $\| \cdot \|_1$ are ℓ_2 and ℓ_1 norms, respectively. Here, $\lambda > 0$ is a penalty (or tuning) parameter and indicates the intensity of the employed penalization. This parameter requires clever selection, which is commonly done using a cross-validation approach. Finally, $0 \leq \alpha \leq 1$ is the mixing parameter: $\alpha = 1$ leads to Lasso, $\alpha = 0$ leads to Ridge and $0 < \alpha < 1$ leads to Elastic Net penalization, respectively. As mentioned earlier, for the sake of simplicity, in this article we select equal weights for the Elastic Net penalty term, i.e., $\alpha = 0.5$.

Appendix B: Performance Evaluation

To assess the performance of a given prediction in both training and test environments, we define the following accuracy measure.

Pearson correlation averaged over environments

$$\text{APCOR}_{\text{Env}}(\hat{\mathbf{Y}}, \mathbf{Y}) = \frac{1}{p} \sum_{j} \rho \left(\hat{Y}_{.,j};\ Y_{.,j} \right).$$

Appendix C: Supplementary Figures

Fig. S1 *Factreg Lasso* regression coefficients for the maize data.

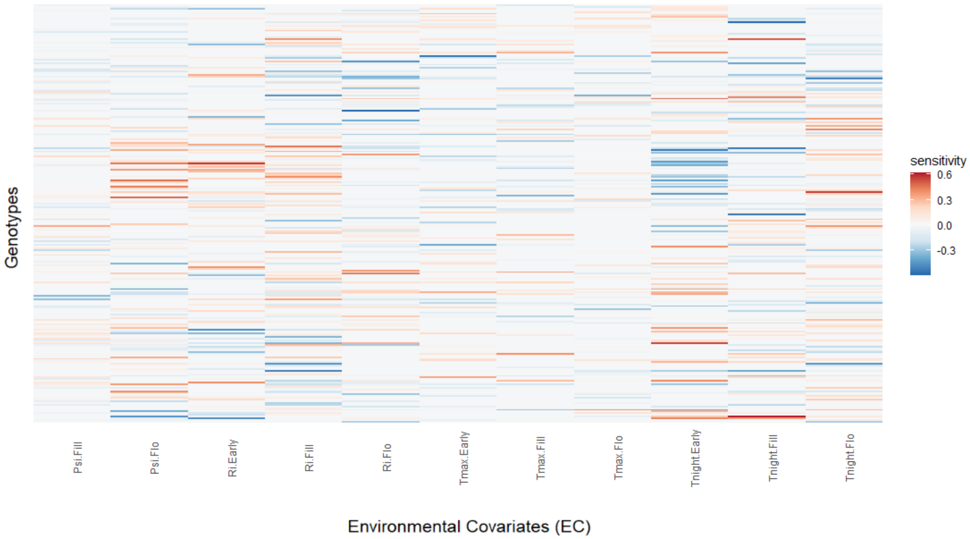

Supplement: Supplementary file 1 — (pdf 239 KB) [file 122_2025_4865_MOESM1_ESM.pdf]
